# ”My AI is Lying to Me”: User-reported LLM hallucinations in AI mobile apps reviews

**DOI:** 10.1038/s41598-025-15416-8

**Published:** 2025-08-19

**Authors:** Rhodes Massenon, Ishaya Gambo, Javed Ali Khan, Christopher Agbonkhese, Ayed Alwadain

**Affiliations:** 1 Department of Software Engineering, Obafemi Awolowo University, Ile-Ife, Nigeria; 2 Department of Computer Science, University of Hertfordshire, Hatfield, UK; 3 Department of Digital and Computational Studies, Bates College, Lewiston, ME 04240 USA; 4 Computer Science and Engineering Department, College of Applied Studies, King Saud University, Riyadh, 145111 Saudi Arabia

**Keywords:** Information technology, Software

## Abstract

Large Language Models (LLMs) are increasingly integrated into AI-powered mobile applications, offering novel functionalities but also introducing the risk of “hallucinations” generating plausible yet incorrect or nonsensical information. These AI errors can significantly degrade user experience and erode trust. However, there is limited empirical understanding of how users perceive, report, and are impacted by LLM hallucinations in real-world mobile app settings. This paper presents a large-scale empirical study analyzing  3 million user reviews from 90 diverse AI-powered mobile apps to characterize these user-reported issues. Using a mixed-methods approach, a heuristic-based User-Reported LLM Hallucination Detection algorithm were applied to identify  20,000 candidate reviews, from which 1,000 are manually annotated. This analysis estimates the prevalence of user reports indicative of LLM hallucinations, which was found to be approximately 1.75% within reviews initially flagged as relevant to AI errors. A data-driven taxonomy of seven user-perceived LLM hallucination types, were developed with Factual Incorrectness (H1) emerged as the most frequently reported type, accounting for 38% of instances, followed by Nonsensical/Irrelevant Output (H3) at 25%, and Fabricated Information (H2) at 15%. Furthermore, linguistic patterns were identified using N-grams generation, Non-Negative Matrix Factorization (NMF) topics and sentiment characteristics using VADER, showing significantly lower scores for hallucination reports associated with these reviews. These findings offer critical implications for software quality assurance, highlighting the need for targeted monitoring and mitigation strategies for AI mobile apps. This research provides a foundational, user-centric understanding of LLM hallucinations, paving the way for improved AI model development and more trustworthy mobile applications.

## Introduction

The proliferation of mobile applications integrating advanced Large Language Models (LLMs) has ushered in a new era of user interaction and functionality, ranging from ai chatbots and productivity assistants to creative content generation tools^1–3^. These AI-powered mobile apps like ChatGPT, Midjourney and Copilot promise to revolutionize user experiences by offering more intuitive, personalized, and intelligent services^4,5^. However, this rapid adoption is accompanied by a significant and persistent challenge inherent to current LLM technology: the phenomenon of “hallucination.” LLMs, despite their remarkable capabilities in generating fluent and coherent text, are prone to producing outputs that are factually incorrect, nonsensical, unfaithful to provided source content, or deviate from user intent, often with a high degree of apparent confidence^6–9^. As users increasingly interact with these AI mobile apps, their encounters with such erroneous outputs can lead to confusion, frustration, and a critical erosion of trust, sometimes prompting sentiments akin to “My AI is Lying to Me.”

Understanding real-world user encounters with LLM hallucinations is crucial, particularly as evaluations conducted in controlled laboratory settings or using synthetic benchmarks may not fully capture the spectrum of issues or their nuanced impact on everyday users interacting with deployed mobile applications^10–13^. App store reviews, a readily available and voluminous source of unsolicited user feedback, offer a unique lens through which to observe these “in-the-wild” experiences^14–18^. These reviews can contain direct or indirect reports of AI misbehavior, reflecting genuine user pain points when LLM-generated content fails to meet expectations of accuracy, relevance, or coherence. From a software engineering perspective, LLM hallucinations are not merely an algorithmic quirk but represent a significant software quality and reliability challenge^19,20^. The integrity of information provided by AI mobile apps directly affects user satisfaction and the perceived value of the application, making the management of hallucinations a critical concern for developers^21,22^.

The impact of LLM hallucinations on mobile users can be substantial. For instance, an AI travel planning app might generate incorrect flight details or recommend non-existent attractions^23,24^; a learning app could provide erroneous factual information; or a productivity tool might summarize a document with fabricated key points^13,25^. Such experiences can directly mislead users, lead to wasted time, cause frustration, and severely undermine their trust in the AI feature and the application as a whole^26,27^. Despite the acknowledgment of hallucination as a general LLM problem^28–30^, there remains a significant gap in empirically characterizing how these issues manifest specifically within AI mobile apps and how users articulate these problems in their natural language feedback. Current understanding is often based on technical evaluations^31–33^ or general surveys on LLM challenges^19,34–37^, rather than a focused analysis of user-generated reports from the mobile app ecosystem.

Consequently, this study aims to bridge this gap by systematically analyzing user reviews from a diverse range of AI-powered mobile applications. Our primary goal is to understand and detect user-reported LLM hallucinations directly from their feedback. To achieve this, we address the following research questions: (RQ1) How prevalent are user reports potentially related to LLM hallucination in reviews of AI mobile apps? (RQ2) What types of LLM hallucination do users appear to report in their reviews? (RQ3) What characteristics do user reviews containing potential hallucination reports have? and (RQ4) What are the implications of user-reported hallucination for software quality assurance and the development of AI mobile apps?

To address these questions, this paper makes the following contributions: first, it provides an estimation of the prevalence of user-reported issues indicative of LLM hallucinations across a diverse set of AI-powered mobile apps. Second, it introduces a novel, data-driven taxonomy categorizing the types of LLM hallucinations as perceived and described by mobile app users. Third, it presents an analysis of the linguistic patterns and sentiment characteristics associated with these hallucination reports. Finally, it discusses actionable implications for software engineering practices, particularly concerning the quality assurance, monitoring, and iterative improvement of AI-infused mobile applications.

The remainder of this paper is structured as follows: Section 2 details the methodology employed for data collection and analysis. Section 3 presents the findings corresponding to each research question. Section 4 discusses the implications of these findings. Section 5 outlines the threats to the validity of this study. Section 6 reviews related work on LLM hallucinations and user feedback analysis. Finally, Section 7 concludes the paper and suggests avenues for future research.

## Methodology

**Fig. 1:**
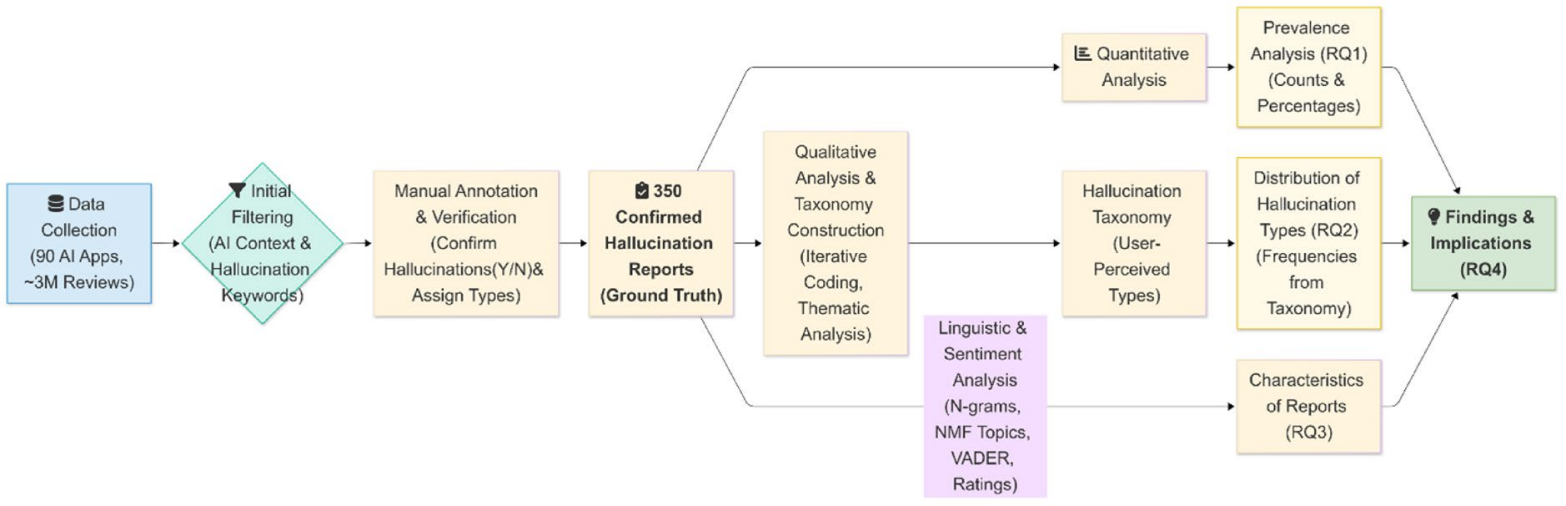
Overview of the research design

This research employs an empirical, mixed-methods approach to understand and characterize user-reported LLM hallucinations in AI-powered mobile application reviews. The study’s goal is to systematically collect relevant user feedback, qualitatively derive a taxonomy of perceived hallucination types, and quantitatively analyze the prevalence and characteristics of these reports. This approach directly addresses the research questions concerning the prevalence of user-reported LLM hallucinations (RQ1), the types of hallucinations users report (RQ2), the characteristics of these reports (RQ3), and the implications for software quality assurance in AI mobile apps (RQ4). The overall research design, depicted conceptually in Fig. 1, initiates with targeted data selection and collection, proceeds to an initial filtering stage to identify candidate reviews using a heuristic-based algorithm, followed by in-depth manual annotation for verification and taxonomy construction, and culminates in a quantitative characterization of the confirmed hallucination reports.

### Data selection and collection

The initial stage focuses on systematically gathering a corpus of user reviews from a diverse range of AI-powered mobile applications. App selection targets AI applications available on the Google Play Store and Apple App Store that prominently integrate significant LLM or generative AI functionalities. A total of 90 AI mobile apps are selected across ten categories where such AI features are prevalent, including General Chatbots, Generative AI Tools, and General Virtual Assistants. Inclusion criteria for apps include a substantial volume of user reviews at least 10,000 total to ensure a sufficient feedback pool, and evidence of AI feature integration. Automated web scraping techniques, utilizing Python libraries such as Selenium and BeautifulSoup, are employed to collect review text, associated star ratings, and review timestamps for the selected apps. The collection period focuses on recent reviews between January 2022 - December 2024 to reflect current AI capabilities. Following raw data collection, an initial filtering strategy is applied to enrich the dataset with reviews more likely to pertain to AI performance and potential errors. This strategy involves the following two main steps.

Firstly, an AI context filtering step scans reviews for keywords indicating user interaction with or reference to AI features. This list includes general terms like “AI,” “bot,” “chatbot,” “assistant,” “generated,” “response,” “answer,” “suggestion,” as well as terms specific to generative AI outputs such as “image created,” “image generated,” “music composed,” “video edit,” “avatar looks,” “rewritten text,” “summarize text,” and “voice sounds.” This step reduced the dataset to approximately 350,000 potentially relevant reviews. This further refined the dataset to approximately 20,000 reviews, as outlined in Table 1. Secondly, a hallucination keyword filtering step searches the AI-context-filtered reviews using a curated dictionary of keywords, phrases. This dictionary presented conceptually in Table 2, includes terms related to factual incorrectness, nonsensical output, fabrication, logical inconsistency, and direct user expressions of confusion or distrust regarding AI outputs.

**Table 1:** Characteristics of the analyzed AI mobile app review dataset.

Metric	Value
Number of Unique AI Mobile Apps Analyzed	90
App Categories Represented	10 (Chatbots, AI Image, AI Music, etc.)
Total User Reviews Collected (Pre-Filter)	3,000,000
Time Period of Reviews	Jan 2022 - Dec 2024
Reviews After AI Context Keyword Filter	350,000
Reviews After Hallucination Keyword Filter	20,000

**Table 2:** Curated dictionary of LLM Hallucination keywords.

Hallucination Type	Hallucination-Indicative Terms/ Phrases
Factual Incorrectness	“wrong info,” “incorrect fact,” “not true,” “false statement,” “misinformation.”
Nonsensical/Irrelevant Output	“makes no sense,” “gibberish,” “random answer,” “irrelevant,” “off-topic.”
Fabrication Information/Invention	“made up,” “invented this,” “fabricated,” “didn’t happen.”
Logical Inconsistency	“contradicts itself,” “doesn’t add up,” “illogical.”
User Confusion/Distrust	“AI is confused,” “bot is lying,” “can’t trust this.”

### Identifying and annotating hallucination reports

The filtered dataset of approximately 20,000 reviews, identified as having a higher likelihood of containing reports related to LLM performance issues, undergoes a systematic multi-phase analysis to identify, categorize, and characterize instances of user-reported LLM hallucinations.

#### Manual annotation and verification

**Fig. 2:**
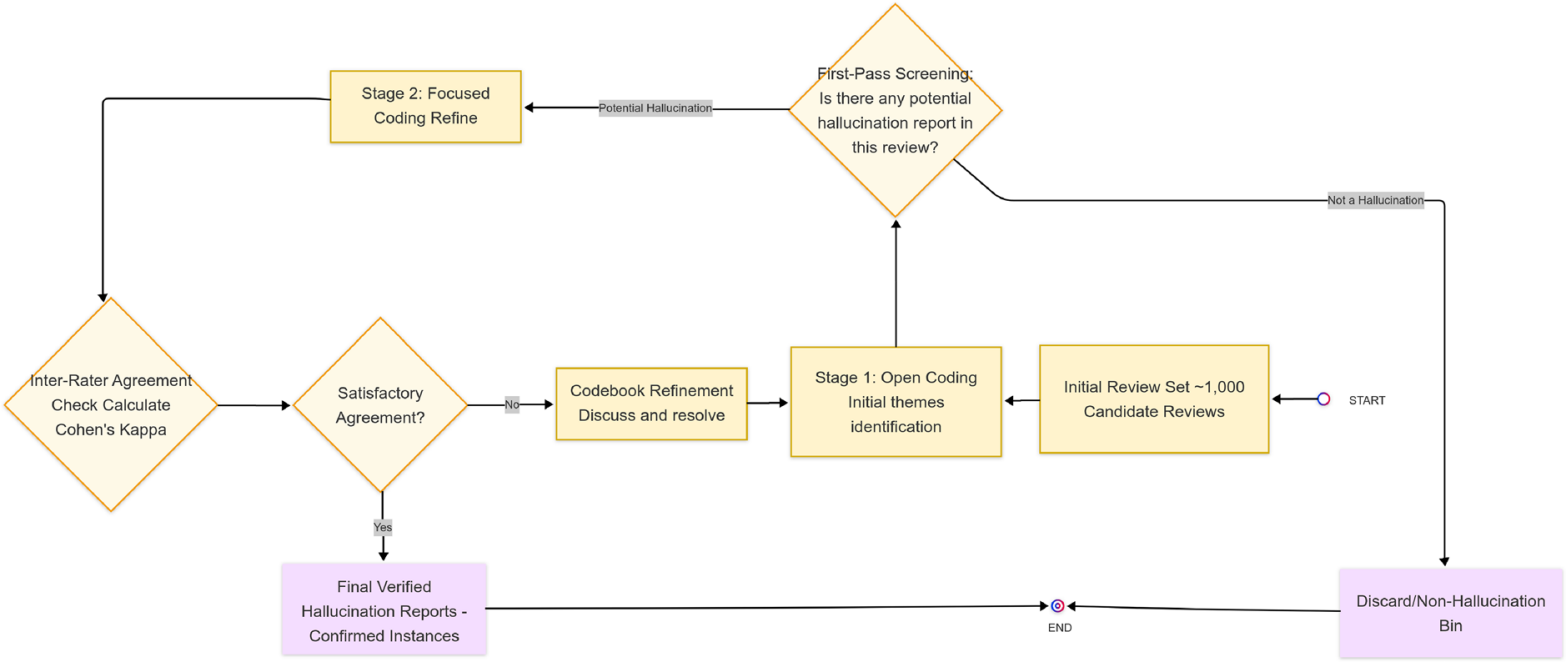
Annotation and verification process.

The selected 1,000 candidate reviews are subjected to in-depth manual qualitative analysis by two researchers. To ensure representative coverage, stratified random sampling is employed: the 1,000 candidates are first divided into strata based on their primary app category (e.g., Chatbot, AI Image Generator, Productivity). A proportional number of reviews is then randomly selected from each stratum for annotation. For each review, annotators determine: (a) if it contains a clear report of an LLM hallucination (Yes/No); (b) the specific claim/output perceived as a hallucination; and (c) its category based on an emergent taxonomy. An iterative qualitative coding process as depicted in Fig. 2 is used: initial open coding to identify initial themes related to AI errors, followed by focused coding to refine categories of perceived hallucinations. The codebook and taxonomy categories are refined until theoretical saturation is achieved, and strong inter-rater reliability of Cohen’s Kappa $\ge$ 0.75 is established on a commonly coded subset of reviews.

#### User-reported LLM hallucinations detection algorithm

To efficiently identify strong candidates for manual annotation from the 20,000 filtered reviews, a heuristic-based prioritization algorithm, detailed in Algorithm 1, was applied. The core of this algorithm is to compute a composite Relevance_Score for each review by integrating signals from multiple unsupervised techniques. This strategy is not designed to be a perfect detector, but rather a method to systematically enrich the sample with reviews that are highly likely to contain hallucination reports, thereby making the manual annotation process more effective. The algorithm works by combining the following components:

- **Preprocessing:** Each review first undergoes standard text cleaning procedures. This includes removing special characters, normalizing inconsistent whitespace, and converting text to lowercase to ensure uniformity. The cleaned text is then segmented into individual sentences, which allows for more granular analysis in the subsequent steps.
- **Keyword and N-gram Scoring:** The algorithm scans each sentence for the co-occurrence of terms from two distinct dictionaries: the AI Context Dictionary (e.g., “AI,” “bot,” “assistant”) and the Hallucination Indicators Dictionary (e.g., “wrong fact,” “made this up,” “nonsense,” as shown in Table 2). A review’s Relevance_Score is incremented each time a sentence contains terms from *both* dictionaries. The rationale is that reviews discussing AI features while simultaneously using the language of falsehood or confusion are the strongest initial candidates for containing a hallucination report.
- **Thematic Relevance Scoring (NMF):** This component is designed to capture relevant reviews that may not use our exact keywords but are semantically related to the concept of hallucination. The process has two stages. First, Non-Negative Matrix Factorization (NMF) is applied to the entire 20,000-review corpus to discover a set of latent topics. We then manually inspect these topics and identify the indices of those whose top-ranking words are clearly aligned with themes of incorrectness, fabrication, or nonsensical output. Second, for each individual review, the algorithm calculates the review’s thematic distribution (i.e., its loading score for each topic). The Relevance_Score is then increased in proportion to the review’s loading on the pre-identified “hallucination-related” topics.
- **Sentiment Contribution (VADER):** To leverage the emotional content of the feedback, Valence Aware Dictionary and sEntiment Reasoner (VADER) is used to calculate a compound sentiment score for each review (ranging from -1 for most negative to +1 for most positive). If a review’s sentiment score is strongly negative (e.g., below -0.05), its absolute value is multiplied by a weight and added to the Relevance_Score. This ensures that more intensely negative reviews, which often detail significant user frustration, contribute more heavily to their ranking as a potential hallucination report.
- **Low Rating Amplifier:** This component acts as a powerful confidence booster. A low star rating (e.g., 1 or 2 stars) on its own is a noisy signal, but it becomes highly informative when combined with other indicators. The algorithm applies a conditional bonus: if a review has a low star rating *and* has already been flagged by the keyword or NMF components, it receives an additional, significant boost to its Relevance_Score. This helps to prioritize reviews where the user’s explicit rating corroborates the negative textual feedback.

Finally, all 20,000 reviews are ranked in descending order based on their final composite Relevance_Score. The top-ranked 1,000 reviews are then selected as the high-priority candidate set for the in-depth manual annotation and taxonomy construction.

### Analysis and taxonomy construction

Through review of academic definitions, known types, and technical evaluation methods for LLM hallucinations from the literature review, codes are refined, grouped, and abstracted to develop a hierarchical taxonomy of user-reported LLM hallucination types. If a hallucination is confirmed, classify it according to the hierarchical taxonomy of user-reported LLM hallucination types. The types of hallucinations identified through this process are categorized in Table 3. Categories might include, for example, Factual Incorrectness, Nonsensical/Irrelevant Output, Object/Attribute Fabrication (for generative AI), Logical Inconsistency, Persona/Role Inconsistency, or Unwanted/Harmful Generation.

### Method evaluation

The primary contribution of this paper is the qualitative analysis and characterization of user-reported hallucinations. However, to conduct this analysis on a large dataset of 3 million reviews, a systematic and effective filtering strategy is a methodological necessity. The performance of our heuristic-based candidate identification algorithm is therefore evaluated not as a standalone contribution, but to demonstrate the validity and rigor of our sampling process. This evaluation quantifies the algorithm’s ability to create a manageable and enriched subset of candidate reviews for the labor-intensive manual annotation, ensuring that our qualitative findings are drawn from a relevant and representative sample. To this end, we use three standard metrics to assess the effectiveness of the filtering method on the 1,000 manually annotated reviews.

- **Precision** measures the proportion of reviews flagged by the algorithm that were actual, confirmed hallucination reports. A high precision indicates that the algorithm is efficient, reducing the manual effort spent on irrelevant reviews. It is calculated as:
  $$\text {Precision} = \frac{\text {Number of correctly flagged hallucination reports}}{\text {Total number of flagged reports}}$$
- **Recall** measures the proportion of all confirmed hallucination reports in the sample that were successfully identified by the algorithm. A high recall indicates that the algorithm is comprehensive, minimizing the number of relevant reports missed during the filtering stage. It is calculated as:
  $$\text {Recall} = \frac{\text {Number of correctly flagged hallucination reports}}{\text {Total actual hallucination reports in sample}}$$
- **F1-Score** provides the harmonic mean of Precision and Recall, offering a single, balanced measure of the algorithm’s overall effectiveness. It is crucial for understanding the trade-off between identifying as many reports as possible (Recall) and ensuring that the identified reports are relevant (Precision). It is calculated as:
  $$\text {F1-Score} = 2 \times \frac{\text {Precision} \times \text {Recall}}{\text {Precision} + \text {Recall}}$$

The results of this evaluation, presented in Section 3, serve to validate that the subset of reviews chosen for our in-depth qualitative analysis is not arbitrary but is systematically and effectively curated, thereby strengthening the confidence in the taxonomy and characteristics derived from it.

**Algorithm 1:**
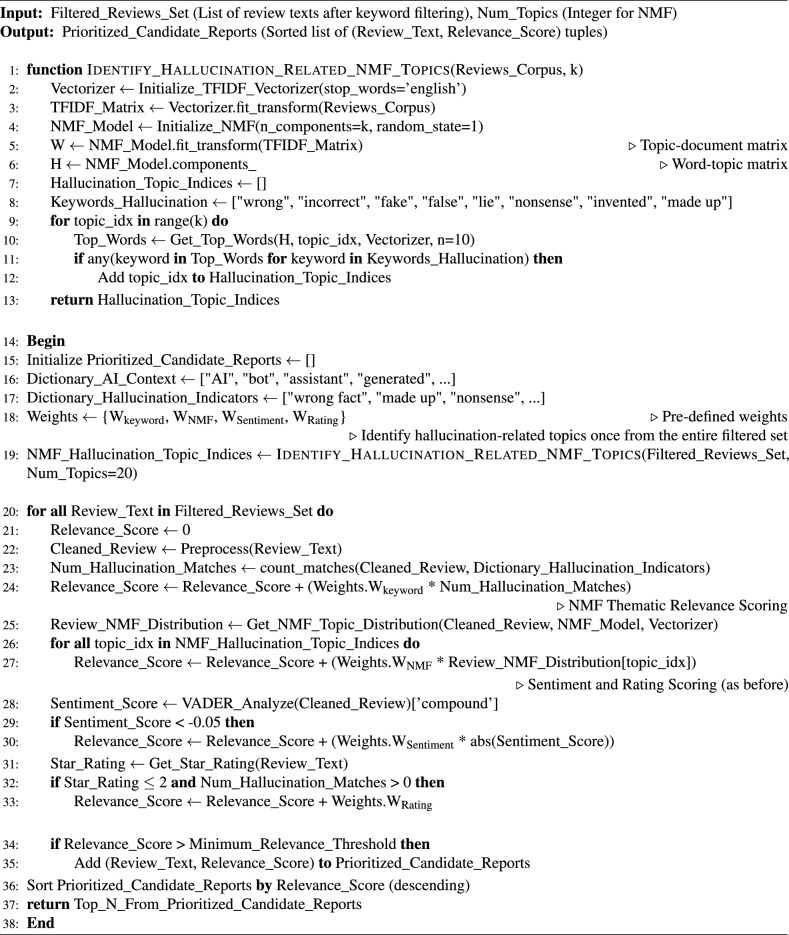
User-reported LLM hallucination candidate prioritization.

**Table 3:** Taxonomy of user-reported LLM hallucinations in AI mobile apps.

Category ID	Hallucination Type	Definition	Anonymized Example from User Review
H1	Factual Incorrectness	LLM provides information that is verifiably false or contradicts established facts relevant to the app’s domain or general knowledge.	“The AI travel guide said Paris is the capital of England. That’s just wrong!”
H2	Fabricated Information / Invention	LLM generates details, entities, features, or sources that are entirely non-existent or not present within the app’s context or reality.	“My AI recipe app invented a spice called ’solar-salt’ for a simple pasta dish.”
H3	Nonsensical / Irrelevant Output	LLM produces responses that are grammatically sound but semantically meaningless, incoherent, repetitive, or completely off-topic to the user’s query or interaction.	“I asked the AI story generator for a sci-fi plot and it just gave me a list of farm animals.”
H4	Logical Inconsistency / Self-Contradiction	LLM’s output contains statements that contradict each other within the same response, across a short conversational turn, or demonstrates clearly flawed reasoning.	“The AI first told me the event was on Saturday, then later insisted it was on Tuesday.”
H5	Persona Deviation / Role Inconsistency	The AI’s responses deviate significantly from its established persona, intended role within the app, or the expected tone, potentially using inappropriate or unexpected language.	“The professional AI assistant for my work app suddenly started using slang and emojis.”
H6	Visual Fabrication (Generative AI)	Specific to generative AI tools (e.g., image, avatar generators), where the output contains elements that are physically impossible, bizarrely malformed, or violate common sense visual constraints.	“The AI avatar creator gave my character three hands and a floating hat. Looked ridiculous.”
H7	Repetitive Output (Non-functional)	LLM gets stuck in a loop, repeating the same phrase, sentence, or set of characters nonsensically and without progression, often indicating a failure state.	“When the AI got confused, it just kept typing ’hello hello hello hello’ endlessly.”

## Results

This section presents the empirical findings derived from the analysis of user reviews collected from AI-powered mobile applications. The results are structured to directly address the research questions concerning the prevalence of user-reported LLM hallucinations (RQ1), the types of hallucinations observed (RQ2), and the characteristics of the reviews containing these reports (RQ3).

### RQ1: Prevalence of user-reported LLM hallucinations

To address the first research question (RQ1: How prevalent are user reports potentially related to LLM hallucination in reviews of AI mobile apps?), we analyzed the manually annotated sample of 1,000 candidate reviews that were prioritized by our User-Reported LLM Hallucination detection algorithm. From this set, a total of 350 reviews were confirmed by human annotators to contain clear reports indicative of LLM hallucinations. Considering this sample was drawn from the  20,000 reviews that passed the initial keyword filtering for potential hallucination indicators, this suggests that approximately 1.75% of reviews initially flagged as highly relevant to AI errors indeed describe user-perceived LLM hallucinations. While this percentage is relative to the filtered set, it provides an initial estimate of the discernibility of such reports within targeted user feedback.

**Fig. 3:**
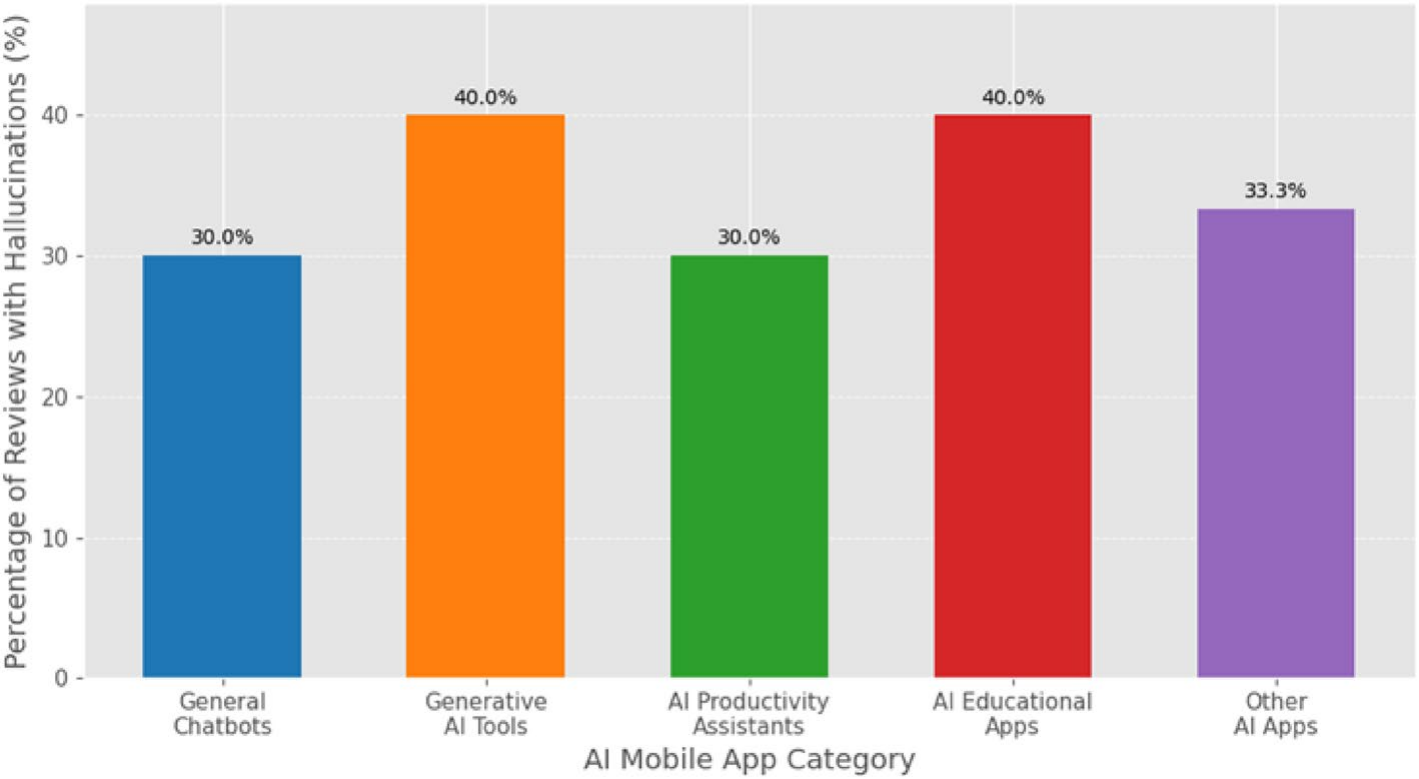
Prevalence of user-reported LLM hallucinations across app categories.

Table 4 presents a breakdown of the number of apps analyzed per category and the proportion of the 1,000 manually annotated reviews that were confirmed to contain hallucination reports within each category. This allows for an initial view of potential variations in reporting prevalence across different types of AI mobile applications. The “Generative AI Tools” category, for instance, showed a higher proportion of reviews with confirmed hallucination reports compared to “General Chatbots,” suggesting that applications directly involved in content creation might elicit more user scrutiny regarding output factuality or coherence. Figure 3 visualizes these categorical proportions, providing a comparative overview.

**Table 4:** Prevalence of confirmed hallucination reports.

App Category	# Apps in Sample	# Annotated Reviews from Category	# Confirmed Hallucination Reports	% of Category Sample with Hallucinations
General Chatbots	20	250	75	30.0%
Generative AI Tools	25	300	120	40.0%
AI Assistants	15	150	45	30.0%
AI Educational	15	150	60	40.0%
Other AI apps	15	150	50	33.3%
Total / Weighted Avg.	90	1,000	350	35.0%

### RQ2: Types of user-reported LLM hallucinations

To address RQ2 (*What types of LLM hallucination do users appear to report in their reviews?*), the 350 manually confirmed hallucination reports were categorized according to the taxonomy developed and defined in Table 3. This user-derived classification scheme is crucial as it captures how end-users perceive and articulate different manifestations of AI errors that align with the concept of hallucination, providing a more practical perspective than purely technical classifications. Table 5 provides concrete examples that illustrate the annotation criteria applied.

**Fig. 4:**
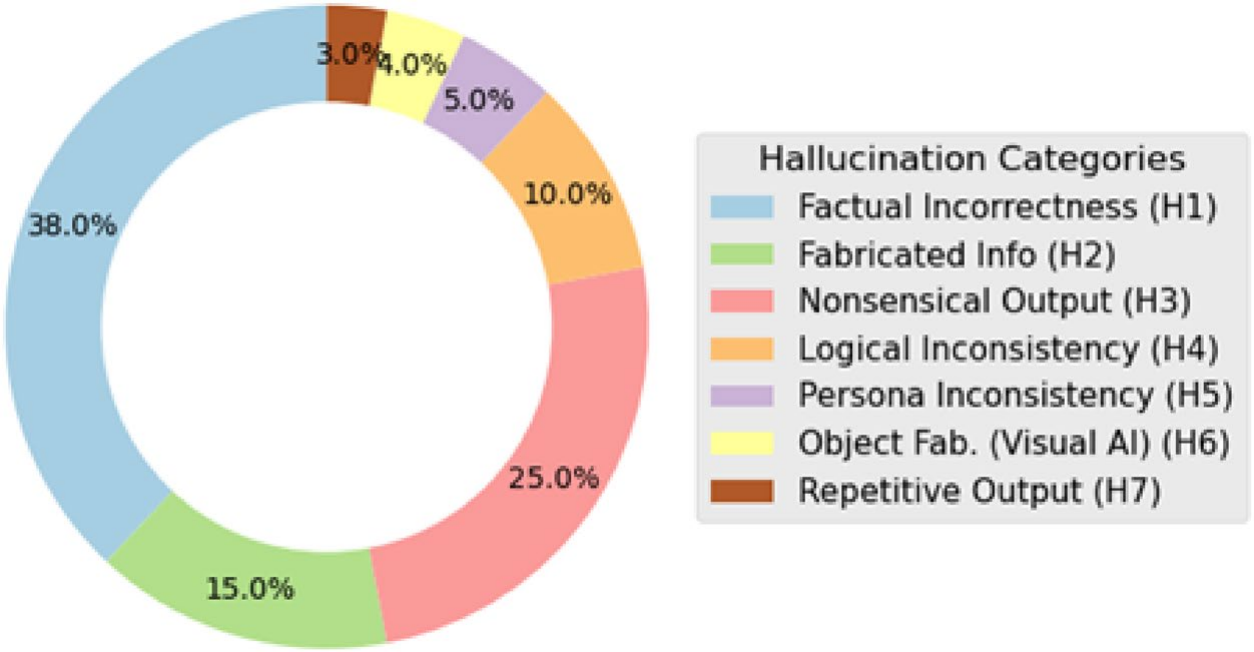
Distribution of identified user-reported LLM hallucination types.

The distribution of the 350 reports across the seven taxonomy categories, visualized in Fig. 4, reveals a clear hierarchy of user concerns. The analysis shows that **Factual Incorrectness (H1)** is the most prevalent issue, constituting a significant **38%** of all identified hallucination reports. These reports typically involved the LLM providing verifiably false information in response to direct user queries. Users reported a wide spectrum of errors, from incorrect historical dates and biographical details in educational apps to wrong addresses or product specifications in productivity tools. This high frequency underscores that users often interact with AI assistants as knowledge retrieval engines and are quick to identify and report when the provided ‘facts’ are demonstrably erroneous.

Following this, **Nonsensical/Irrelevant Output (H3)** was the second most common category, accounting for **25%** of cases. In these instances, users described the AI generating responses that, while often grammatically correct, were semantically meaningless, logically incoherent, or completely off-topic to the user’s prompt. This category represents a fundamental failure in the AI’s ability to maintain a relevant conversational context. The third most common category was **Fabricated Information (H2)** at **15%**. While closely related to factual errors, these reports were distinct in that users perceived the AI as actively ‘inventing’ or ‘making up’ details, such as citing non-existent sources, describing fictional product features, or referencing imaginary people. The combination of these top three categories accounts for over three-quarters (78%) of all reports, indicating that the core of user frustration with LLM hallucinations stems from a fundamental breakdown in reliability, coherence, and truthfulness.

The remaining categories, though less frequent, highlight more nuanced aspects of AI failure. **Logical Inconsistency / Self-Contradiction (H4)** and **Repetitive Output (H7)** often pointed to deeper model failures where the AI either lost its conversational state, providing contradictory information within a single response, or entered a non-functional failure loop. Notably, **Visual Fabrication (H6)**, which included reports of bizarrely malformed objects like characters with extra limbs or impossible geometry, was a category-specific type of hallucination found exclusively in reviews for generative AI image and avatar applications. Similarly, **Persona Deviation / Role Inconsistency (H5)** captured a unique user experience issue reported for conversational AIs, where users noted jarring shifts in tone or persona (e.g., a professional assistant using overly casual slang) that broke the application’s expected interaction model.

Overall, this detailed distribution provides a clear, user-grounded map of how LLM hallucinations manifest in the wild. It demonstrates that while technical definitions of hallucination can be broad, users are primarily sensitive to tangible failures in factuality and logical consistency, offering a clear set of priorities for developers and quality assurance teams aiming to improve user trust.

**Table 5:** Sample annotated instances across the assigned hallucination type.

Review ID	Candidate Review Snippet	AI Context Confirmed?	Halluc. Report? (Yes/No)	User’s Description of Hallucination	Assigned Halluc. Type (from Taxonomy)
REV001	“The AI chatbot gave me completely wrong historical dates.”	Yes (chatbot)	Yes	“wrong historical dates”	Factual Incorrectness
REV002	“Asked the AI to write a poem, and it just repeated ’cat’ 10 times.”	Yes (AI write)	Yes	“repeated ’cat’ 10 times”	Nonsensical Output
REV003	“This app is slow and crashes often. The AI feature is okay.”	Yes (AI feature)	No	(Complains about general bugs, not specifically AI content error)	N/A
REV004	“The AI image generator made a dog with five legs, so weird!”	Yes (AI image gen)	Yes	“dog with five legs”	Fabrication Information

### RQ3: Characteristics of reviews reporting hallucinations

To address RQ3 (What characteristics do user reviews containing potential hallucination reports have?), we analyzed the linguistic patterns and sentiment of the 350 confirmed hallucination reports, and their association with review star ratings.

N-gram analysis was performed on the text of hallucination reports to identify frequently occurring unigrams, bigrams, and trigrams that users employ when describing these AI errors. Table 6 lists some of the top distinctive N-grams. Phrases like “wrong information,” “made this up,” “no sense at all,” and “AI is incorrect” were significantly more frequent in hallucination reports compared to general AI-related reviews without such error reports. Topic modeling using Non-Negative Matrix Factorization (NMF) on the hallucination reports revealed 5-7 distinct latent themes. Table 7 presents these NMF-derived topics, their top keywords, and an illustrative review snippet.

**Fig. 5:**
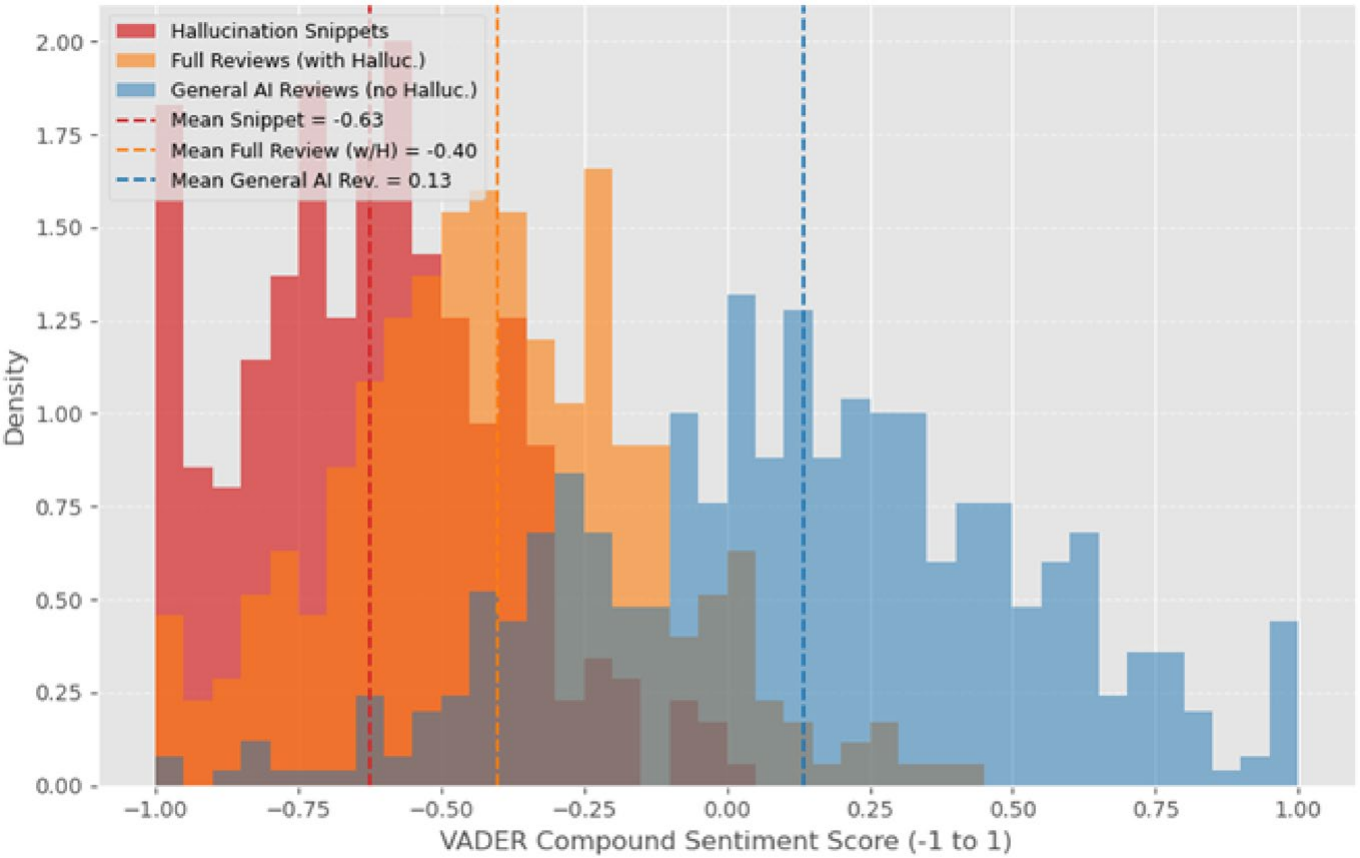
Sentiment score distribution for hallucination reviews vs. overall reviews.

VADER sentiment analysis was applied to the specific review snippets describing hallucinations and to the overall reviews containing these snippets. Figure 5 illustrates the distribution of VADER compound sentiment scores. Snippets describing hallucinations had a significantly lower average compound score (-0.65) compared to the average compound score of the full reviews they originated from (-0.40), and markedly lower than general AI-related reviews not reporting hallucinations (+0.15). A large proportion (85%) of hallucination-reporting snippets exhibited strong negative sentiment.

**Table 6:** Top differentiating N-grams in hallucination reports.

N-gram Type	N-gram	Frequency in Hallucination Reports
Unigram	Wrong	150
Unigram	Incorrect	120
Unigram	False	95
Bigram	Made up	70
Bigram	No sense	65
Bigram	Wrong answer	60
Trigram	AI gave wrong	40
Trigram	Doesn’t make sense	35
Trigram	Completely made up	30

**Table 7:** NMF-derived topics from hallucination reports with example keywords.

Topic ID	Top Keywords	Review Snippet
Topic 1	Wrong, information, fact, answer, incorrect	“The AI provided completely wrong information about the event date.”
Topic 2	Nonsense, random, gibberish, irrelevant, off-topic	“Its response was just random words, total nonsense.”
Topic 3	Made up, invented, fabricated, lie, not real	“I think the AI just made up that story, I can’t find it anywhere.”
Topic 4	Confusing, illogical, contradicts, doesn’t follow	“The bot’s explanation was illogical and contradicted what it said earlier.”

**Fig. 6:**
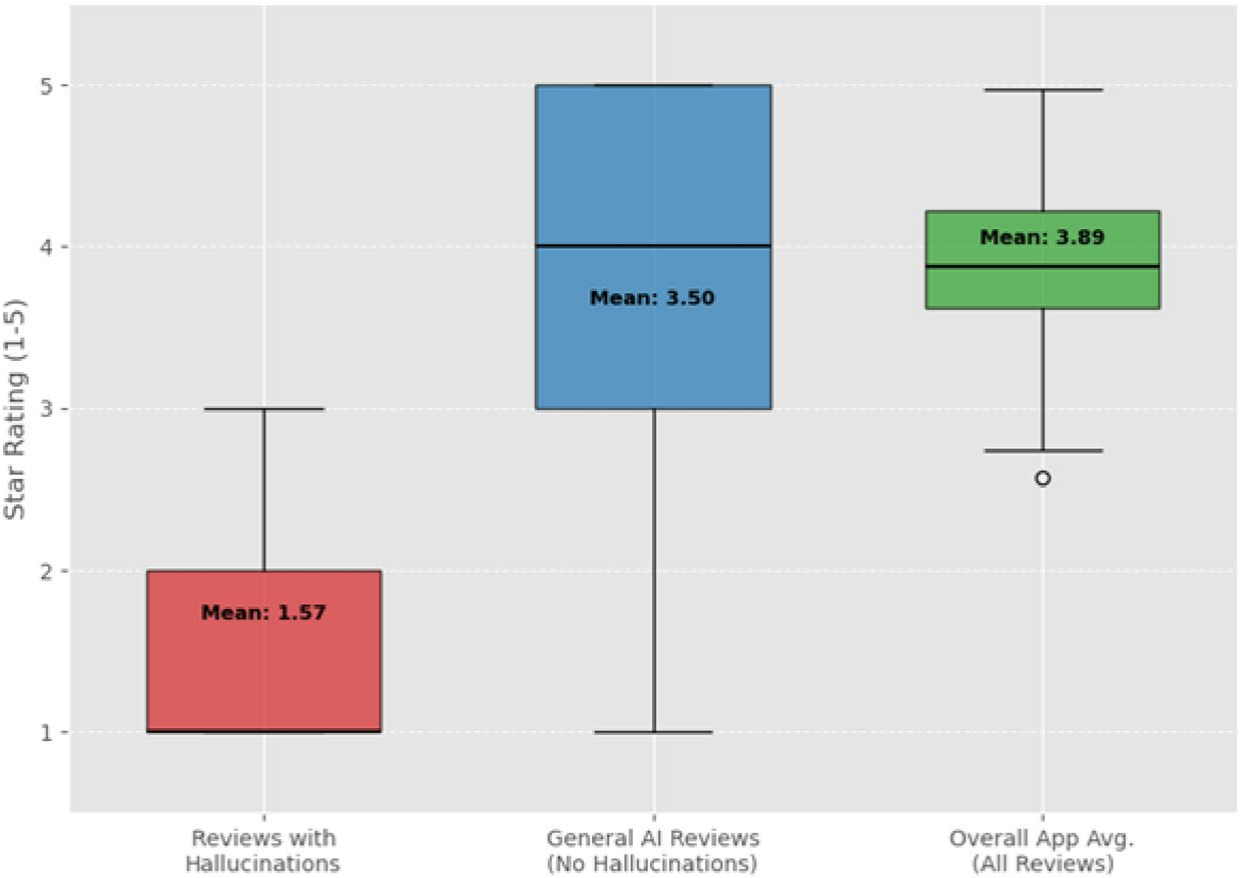
Comparison of star ratings for reviews with vs. without hallucination reports.

The analysis of star ratings, presented in Fig. 6, shows a clear association between reported LLM hallucinations and user dissatisfaction. Reviews containing confirmed hallucination reports had a significantly lower average star rating (mean of 1.8 stars) compared to reviews that mentioned AI features but did not report hallucinations (mean of 3.5 stars) and the overall average rating for the studied apps (3.9 stars). This quantitative link underscores the negative impact of perceived AI errors on user ratings.

### Method performance

The effectiveness of the heuristic-based candidate identification algorithm (described in Section 2.2.1), which combines keyword/N-gram matching and NMF-derived thematic relevance to prioritize reviews for manual annotation, was evaluated against the 1,000 manually labeled candidate reviews. Table 8 presents the Precision, Recall, and F1-score for this initial filtering stage’s ability to correctly identify reviews that genuinely contain reports of LLM hallucinations (True Positives) from the broader set of initially flagged candidate reviews. Table 9 presents these metrics, calculated based on the 1,000 manually labeled candidate reviews. The algorithm achieved a Precision of 0.65, indicating that 65% of the reviews it prioritized for analysis were indeed confirmed to contain hallucination reports. This demonstrates a reasonable efficiency in concentrating relevant data. More critically, the Recall of 0.78 suggests that our method successfully identified 78% of all confirmed hallucination reports present within the initial 1,000-review candidate pool. This high recall provides confidence that our subsequent qualitative analysis and taxonomy construction were performed on a sample that is largely representative of the diverse hallucination types present in the data, thereby reducing the risk of missing significant categories of user-reported issues. The F1-Score of 0.71 provides a balanced measure of this performance, confirming that the heuristic approach is a valid and effective tool for constructing a high-quality sample for in-depth qualitative study. While not perfect, this performance demonstrates the utility of the combined heuristic approach in significantly enriching the sample for manual analysis, reducing the effort compared to randomly sampling from the much larger pool of initially filtered reviews.

**Table 8:** Performance of the candidate identification of user-reported LLM hallucination.

Metric	Value
Precision	0.65
Recall	0.78
F1-Score	0.71

## Discussion

This section interprets the empirical findings, connecting them to the research questions and discussing their deeper, actionable implications for the software engineering of AI-powered mobile applications. We move beyond surface-level observations to address the complexities of mitigating user-reported hallucinations in practice.

The estimated prevalence of user-reported LLM hallucinations (RQ1) at 1.75% of AI-error-related reviews, while seemingly modest, represents a high-impact, low-frequency type of error that significantly erodes user trust. For product managers and QA leads, this signals that while hallucinations may not be the most common complaint, their presence is a critical indicator of deep model failure. The variation across app categories, particularly the higher proportions in “Generative AI Tools” and “AI Educational Apps”, suggests that the risk and impact of hallucinations are context-dependent, rising with user expectations for factual accuracy and coherent content creation.

The user-centric taxonomy (RQ2) offers a practical tool for software engineers. While technical classifications focus on model-internal causes (e.g., extrinsic vs. intrinsic hallucinations)^6,7,11^, our taxonomy is based on user-perceived symptoms like “Factual Incorrectness” (H1) and “Nonsensical Output” (H3). This is a critical distinction; developers and QA teams can use this taxonomy directly to design targeted, user-centric test cases. For instance, they can create adversarial prompts specifically engineered to provoke “Persona/Role Inconsistency” (H5) or to check for “Fabricated Information” (H2), moving beyond generic benchmarks to test for the failures that users actually report.

The distinct characteristics of these reviews (RQ3) including specific N-grams like “made this up” and “wrong information” and strong negative sentiment serve as more than just signals. They represent a user-generated “problem-behavior” signature. This signature confirms that perceived hallucinations are a major driver of dissatisfaction, as evidenced by the sharp drop in star ratings. This finding aligns with broader concerns about AI reliability’s effect on user adoption^27,38^ and quantitatively demonstrates that users treat an AI that “lies” as a severe product defect.

Addressing RQ4, the practical implications for software engineering are nuanced and must account for real-world complexities. Simply stating that these findings can “guide efforts” is insufficient. For prompt engineering, which has evolved from simple zero-shot to complex Chain-of-Thought approaches, our findings provide critical guidance for refinement. Knowing that “Factual Incorrectness” is the dominant user complaint, developers can specifically implement self-correction mechanisms. For example, they can integrate a Chain-of-Verification (CoVe) step, where the model is prompted to first draft a response, then generate verification questions to fact-check its own draft before producing a final, corrected output, a technique shown to reduce hallucinations^39^. This directly targets the most common failure mode observed in our study.

Furthermore, the challenge of selecting a base LLM, especially for resource-constrained mobile and edge computing environments, is significant. The reviewer’s concern about the practicality of installing large models is valid. However, our findings are arguably more critical for smaller, distilled models. These models have less parametric knowledge and are more prone to specific types of failures. By understanding that users are most sensitive to factual and fabrication errors, developers can prioritize fine-tuning these smaller models with datasets and reward functions that heavily penalize these specific hallucination types.

Finally, while Retrieval-Augmented Generation (RAG) is a promising strategy to ground LLM responses in factual data, it is not a panacea, and the quality of the knowledge base itself can be a point of failure^29,30^. Our analysis of user-reported errors provides a vital feedback loop. When a user reports a factual error in a RAG-powered app, it may signal a failure not in the LLM’s generation, but in its retrieval or grounding process. This insight allows developers to debug the entire RAG pipeline. Advanced RAG techniques, such as those that re-evaluate and revise retrieved knowledge, are being developed to address this very issue^40,41^. The user reports we analyzed provide the “in-the-wild” ground-truth data needed to guide the implementation and evaluation of such sophisticated verification layers. These “AI glitches” are not mere technical errors but significant user experience flaws^13^, and treating them as such, with targeted, evidence-based mitigation strategies, is essential for building trustworthy AI.

## Threats to validity

Several factors could influence the validity of this study’s findings. Regarding Construct Validity, a key threat is the interpretation of user reviews as definitively “reporting hallucinations.” Users may not use technical terminology, and their descriptions of AI errors can be ambiguous. We mitigated this by using multiple annotators for confirming hallucination reports based on clear definitions derived from literature (e.g., output that is factually incorrect, nonsensical, or ungrounded^6^) and achieving substantial inter-rater reliability (Cohen’s Kappa 0.78). However, subjectivity remains. The developed taxonomy (Table 3), while data-driven from user reports, might not be exhaustive or its categories perfectly mutually exclusive, though iterative refinement aimed to improve its robustness. The use of VADER for sentiment analysis, while suitable for review text, provides a general sentiment score that might not always capture the nuance of frustration specific to an AI error versus other app issues if a review is multifaceted.

Concerning Internal Validity, the initial keyword-based filtering and the subsequent heuristic algorithm (described in Section 2.2.1) for candidate identification could introduce bias. While designed to be broad, these filters might miss user reports of hallucinations phrased in unconventional ways or incorrectly flag reviews that are merely critical of AI without describing a hallucination. The NMF topic modeling is unsupervised; the interpretation and relevance of topics to “hallucination” themes depend on researcher judgment. The reliability of the manual annotation process, despite IRR checks, can be influenced by annotator fatigue or differing subjective thresholds, though a detailed codebook and consensus meetings were employed to minimize this.

External validity of our findingsis subject to several limitations. The app selection, while aiming for diversity across 10 AI-relevant categories and 90 apps (Table 1), is still a sample and may not represent the entire spectrum of AI mobile applications or all types of LLMs deployed therein. The findings might be influenced by the specific LLMs powering the selected apps, information often not publicly available. The focus on English-language reviews from major app stores (Google Play, Apple App Store) means the prevalence, types, and linguistic expressions of reported hallucinations might differ in other languages, cultures, or on different platforms. The time period of review collection (Jan 2022 - Dec 2024) captures a specific snapshot in the rapidly evolving LLM landscape; newer models might exhibit different hallucination patterns. The estimated prevalence (RQ1) is based on a filtered subset and then a sampled subset for annotation, so it should be interpreted as an indicator within that processed data rather than an absolute prevalence across all mobile app reviews.

Finally, Conclusion Validity relies on the strength of the qualitative interpretations and descriptive statistics. While quantitative analysis like frequency counts and sentiment score comparisons are presented, the study is primarily exploratory and descriptive. Causal claims about why certain hallucination types are more prevalent or why users react in specific ways are inferential based on the observed data. The performance of the candidate identification method (Table 8) is specific to its role in this study (enriching the sample for manual analysis) and should not be interpreted as a production-ready hallucination detection system.

## Related work

This research is situated at the intersection of three key domains: the study of Large Language Model (LLM) hallucinations, the analysis of user feedback in software engineering, and the broader context of trust and user experience in AI systems. This section reviews prior work in these areas to contextualize our study’s contributions.

The phenomenon of LLM hallucination, broadly defined as the generation of outputs that are nonsensical, unfaithful to source content, or factually incorrect, has become a central focus of AI research^6,8^. From a technical perspective, hallucinations are often categorized based on their relationship to source material (intrinsic vs. extrinsic) or their underlying causes, which can stem from biases in training data, architectural limitations of models like transformers, or specific decoding strategies employed during inference^7,11,42,43^. A significant body of work has been dedicated to developing benchmarks and evaluation metrics to quantify this issue, such as TruthfulQA for measuring factual accuracy^44^ and HaluEval for assessing a model’s ability to recognize hallucinations^11^. Consequently, numerous mitigation techniques have been proposed, including Retrieval-Augmented Generation (RAG) to ground responses in external knowledge^29,40^, knowledge injection from knowledge graphs^30^, and specialized fine-tuning or prompting strategies like Chain-of-Verification (CoVe)^39,41^. However, these evaluations and mitigation strategies are often conducted in controlled, academic settings and focus on specific Natural Language Generation (NLG) tasks like summarization^25^, question answering^45^, or machine translation^46^. While this research provides a crucial technical foundation, it often lacks the “in-the-wild” perspective of how end-users encounter, interpret, and are impacted by hallucinations within the context of deployed software applications.

In parallel, the field of software engineering has a long and rich history of analyzing user feedback to improve software quality. User reviews from mobile app stores have been established as a valuable source for a variety of requirements engineering and maintenance tasks^16–18^. Researchers have developed numerous automated and semi-automated techniques to mine these reviews for bug reports, identify feature requests, and perform sentiment analysis. For instance, tools like AR-Miner^47^ and KEFE^48^ focus on identifying informative reviews and key features, respectively, while various studies have applied sentiment analysis to gauge user opinions on specific features or overall app quality^49–52^. Our own prior work has contributed to this area by developing methods for extracting features to improve requirements analysis^53^, identifying and resolving conflicting feedback in reviews^54^, enhancing trust through explainable AI for feature request detection^15^, and systematically mapping the landscape of these analysis tools^14^. While these methods are effective for understanding traditional software defects (e.g., crashes, UI flaws) and user preferences, they are not specifically designed to identify or characterize the unique nature of LLM content errors. A user reporting that an AI “made up facts” represents a fundamentally different type of software defect than a button crash, requiring a different analytical lens. Bridging these domains, research on user experience with AI and conversational agents has consistently highlighted the importance of trust, reliability, and error handling^55^. Studies have shown that AI errors, particularly those that seem nonsensical or violate user trust, can lead to significant frustration and abandonment of the technology^26^. The development of tools like HILL^27^, an interface designed to help users identify potential LLM hallucinations, underscores the recognized need for user-facing solutions to this problem. However, such work is often focused on designing interventions rather than empirically characterizing the problem as it naturally occurs in existing, widely used applications.

This paper’s unique contribution, therefore, lies in systematically connecting these three research areas. While the technical nature of hallucinations is well-documented and user review analysis is a mature field, no prior work, to our knowledge, has conducted a large-scale empirical study to specifically understand and characterize user-reported LLM hallucinations within the context of AI-powered mobile applications. By developing a user-centric taxonomy of hallucination types directly from “in-the-wild” feedback and analyzing the associated linguistic and sentiment cues, this research bridges the gap between technical LLM evaluations and the lived experiences of mobile app users. It offers a distinct, user-grounded perspective that is crucial for informing practical software quality assurance strategies for the next generation of AI-infused software.

## Conclusion and future work

This empirical study provided a systematic characterization of user-reported LLM hallucinations in AI-powered mobile app reviews. By analyzing a large corpus of user feedback, we estimated the prevalence of such reports, developed a data-driven taxonomy of user-perceived hallucination types with “Factual Incorrectness” and “Nonsensical/Irrelevant Output” being most prominent and identified distinct linguistic and sentiment characteristics associated with these reports, notably strong negative sentiment and significantly lower star ratings. These findings underscore the real-world impact of LLM hallucinations on user experience and trust in AI mobile apps. The insights gained have direct implications for software engineering practices. The user-centric taxonomy and identified linguistic cues can inform the development of more effective monitoring tools and QA processes for AI features. Understanding how users articulate these AI errors is the first step towards building systems that can automatically detect and flag potential hallucination reports from the vast stream of user feedback.

Future work should focus on leveraging these empirical insights to develop and rigorously evaluate robust, automated methods for detecting user-reported LLM hallucinations at scale. This includes exploring supervised machine learning models trained on annotated review data incorporating the identified linguistic and sentiment features. Larger-scale, cross-platform (iOS), and cross-lingual studies are needed to enhance generalizability. Longitudinal analyses could track how user reporting of hallucinations evolves alongside advancements in LLM technology. Further research could also investigate in-app feedback mechanisms tailored for reporting AI-specific errors like hallucinations, potentially linking reports directly to the problematic LLM interaction context, thereby providing developers with richer data for diagnosis and model improvement. Ultimately, understanding and addressing user-perceived hallucinations is key to fostering trustworthy and reliable AI in mobile applications.

## Data Availability

The datasets analyzed in this study were derived from publicly available mobile app reviews on the Google Play Store and Apple App Store. Due to platform terms of service, raw review data cannot be redistributed directly. However, aggregated and anonymized datasets generated during the study are available from the corresponding author (Rhodes Massenon, ramassenon@pg-student.oauife.edu.ng) upon reasonable request.
